# Analysis of the conflicts of interest disclosed by the program reviewers of the scoliosis research society (SRS) congresses, 2010-2014

**DOI:** 10.1371/journal.pone.0204993

**Published:** 2018-10-11

**Authors:** Carlos Barrios, Joaquín Alfonso, José Miguel Lloris, Eduardo Hevia, Jesús Burgos

**Affiliations:** 1 Institute for Research on Musculoskeletal Disorders, Valencia Catholic University, Valencia, Spain; 2 Department of Surgery, Valencia University School of Medicine, Valencia, Spain; 3 Spine Surgery Unit, Hospital La Fraternidad-Muprespa, Madrid, Spain; 4 Division of Paediatric Orthopaedics, Hospital Ramón y Cajal, Madrid, Spain; Johannes Gutenberg Universitat Mainz, GERMANY

## Abstract

**Background:**

Conflicts of interest (COI) between industry and surgeons frequently introduce biases into surgical research. The abstracts submitted for presentation in scientific congresses are usually vetted for any indication of commercial bias. Members of review program committees regularly have recognized qualifications, and therefore certain COI are unavoidable. This study aims to determine the prevalence and magnitude of possible COI among those responsible for the selection of presentations at two important international conferences on spine surgery during a five-year period.

**Methodology:**

COI declarations by those responsible for the final programs of the annual SRS (Scoliosis Research Society) and IMAST (International Meeting of Advanced Spine Technologies) conferences from 2010 to 2014 were collected and analyzed from data published by the corresponding scientific programs. The SRS’s disclosure index did not contain financial amounts; therefore, this aspect could not be analyzed.

**Results:**

Five scientific committees and 117 members (76 individuals) were studied. Of these 76, 41 (53.9%) participated in more than one conflict of interest (>1 COI). Scientific committee members were from 11 countries across 4 continents, but most were from the Unites States (76.9%). Of the 117 program reviewers, 65.8% declared >1 COI and 34.2% reported no COI. The 77 program reviewers who disclosed a potential COI declared a total of 273 COI (mean = 3.54 COI/member). Overall, 36.0%, 26.1%, 10.7%, and 10.7% of the COI corresponded to consultancies, research funds, bureau participation, and advisory board panel participation, respectively. Stockholder reimbursement corresponded to 8.8% of the disclosed COI, and financial or material support were mentioned in 7.4% of COI. Among the COI disclosures, 55 companies were mentioned, and 5 of the top 10 companies involved in spinal device markets were responsible for 65.2% of the COI.

**Conclusions:**

More than two thirds of the members of the SRS and IMAST scientific committees reported COI. Consultancies and research grants account for two thirds of these. Most of the grants and major COI are related to the five companies leading the spinal implant market.

## Introduction

Links between the medical device industry and surgeons are crucial for the development of new therapeutic surgical procedures. In recent decades, the role of industry support in different areas of surgical research has expanded significantly; further, support is now directed toward individuals instead of institutions [1]. These facts are widely acknowledged but remain a source of divisive debate.

Orthopedic surgery is heavily dependent on implants that are often designed, improved, or developed by surgeons in conjunction with industry. It is relatively common for surgeons to hold or participate in the patent of a device because they have a particular interest in its use. These close bonds between industry and surgeons could be a source of potential bias in clinical research and may affect both scientific presentations at clinical meetings and published data [2–4]. A recent study demonstrated significant associations among funding sources, study outcomes, and levels of evidence in spinal research [5], as a large proportion of industry-funded research has been shown to provide level IV evidence and report favorable outcomes.

All orthopedic societies require conflict of interest (COI) disclosure statements from contributors before any presentation or discussion of research results at their scientific meetings, and this information is published in the conference proceedings. Indeed, some surgical specialties in orthopedics have already released audits of the COI disclosures at their congresses [2,4,6].

In spinal surgery, the policy concerning COI disclosures for authors varies significantly. For example, the self-reported disclosure information of authors attending three major annual spine conferences in 2008 (those of the North American Spine Society [NASS], Cervical Spine Research Society [CSRS], and Scoliosis Research Society [SRS]) has been compiled and analyzed, and discrepancies or irregularities in the COI disclosures oscillated from 9% among authors whose data were listed at the NASS and SRS conferences to 51% among authors who presented at the NASS and CSRS meetings [7].

Within orthopedics, the SRS is a leading provider of policies to improve transparency and clarify the relationships between surgeons and industry. The SRS’s website provides the following information to authors: “It is the policy of SRS to ensure balance, independence, objectivity, and scientific rigor in all of their educational activities. In accordance with this policy, SRS identifies conflicts of interest with instructors, content managers, and other individuals who are in a position to control the content of an activity. Conflicts are resolved by SRS to ensure that all scientific research referred to, reported, or used in a continuing medical education (CME) activity conforms to the generally accepted standards of experimental design, data collection, and analysis. Complete faculty disclosures will be included in the final program” [8].

The process of selecting abstracts for presentation at different scientific meetings is a challenge for medical societies. Abstract presentations at SRS meetings are CME accredited activities, and it is therefore essential that submissions be free from commercial influence. Before presentation, all contributions must clear a review by the SRS CME Committee to ensure that no bias issues are included in the final program. In addition, the SRS strongly recommends that authors use generic descriptions of their instrumentation or techniques, with no specific names of implants or surgical devices.

Both abstracts submitted for presentation in scientific congresses and manuscripts submitted for publication in journals are usually vetted for indications of commercial bias. However, members of review program committees and journal review boards regularly have recognized qualifications, and therefore certain COI are unavoidable. Recently, a study analyzed the prevalence and financial magnitude of potential COI among the editorial board members of five leading spinal journals [9]. The percentage of COI disclosure statements among editorial board members varied broadly across journals, ranging from 39% to 71% [9]. This study confirmed that COI in editorial boards might create bias in the peer review process [9]. Previous studies have not, however, analyzed COI among medical associations’ program review committee members.

The first purpose of this study was to determine the prevalence and magnitude of COI reported by members of the review program responsible for the selection of works to be presented at two prestigious international congresses of spinal surgery over a five-year period: the SRS Annual Meeting and the International Meeting of Advanced Spine Technologies (IMAST). Interestingly, review of abstracts submitted to these two meetings is entrusted to a single committee. This fact makes analysis of COI disclosures among committee members even more interesting from merely an observational perspective. The second objective was to measure the type of and variability in COI reported by review committee members and to examine members’ financial relationships with leading medical device companies.

## Materials and methods

### Study design

COI disclosures of members of the review committee responsible for the final program of the annual congresses of the SRS and IMAST during the years 2010–2014 were analyzed. Data were collected from the disclosure index published in the final program of each congress. These meetings were chosen because of their exclusive focus on spinal topics, their single review committee, and the availability of disclosure statement indexes. Disclosure for these meetings was mandatory not only for authors but also for committee and board members.

As per the SRS policy concerning COI, disclosures were categorized according to one of the following: (1) nothing to disclose/no conflict reported and (2) potential conflict of interest. Relationships disclosed were categorized as follows: (a) grant/research support; (b) consultant; (c) stock/shareholder (self-managed); (d) speakers bureau; (e) advisory board or panel; (f) salary/contractual services; and (g) other financial or material support (royalties, patents, etc.). The disclosure indexes of the SRS and IMAST do not contain the financial amount per disclosure; therefore, this aspect could not be analyzed.

Consultant fees, stock options, and royalties were considered major financial COI [2]. To be a member of a speakers bureau, advisory board, or advisory panel and/or to receive a salary or other contractual services were considered minor COI. Research grants were considered separately. Financial relationships with six leading orthopedic/spinal medical device companies mentioned in disclosure statements were also analyzed [10].

### Data analysis

The total number of individuals reporting COI and the types of COI were tabulated. COI frequencies were calculated for each year. As one of the main objectives of this study was to analyze the ratio and type of COI of those reviewers who declared COI, this group was subsequently analyzed more in detail. Statistics were calculated on the basis of reviewer participation and not on individuals, since the same individual could participate multiple years in the Program Committee and the COI might change. Data for all measured variables were examined using descriptive statistical analysis. Contrast analyses were performed using Fisher’s exact test and a Mann–Whitney *U* test. The level of significance was set at *p*<0.05.

## Results

A total of 117 program reviewers of 5 scientific program committees were identified. These corresponded to 76 unique individuals, of whom 41 (53.9%) participated in more than one committee (20 in 2, 11 in 3, 10 in 4 or more). Subsequent statistics will be done in the scientific committee members (117), even if they can correspond to participations of the same individual, since their COI could have changed from time to time. Scientific committee members were from 11 countries, but most were from the United States (90 of 117, 76.9%). Twelve were from the United Kingdom, Spain, Germany, and Turkey; six were from Japan, Singapore, and Malaysia; five were from Egypt; three were Canadian; and one was from Australia.

**Fig 1:**
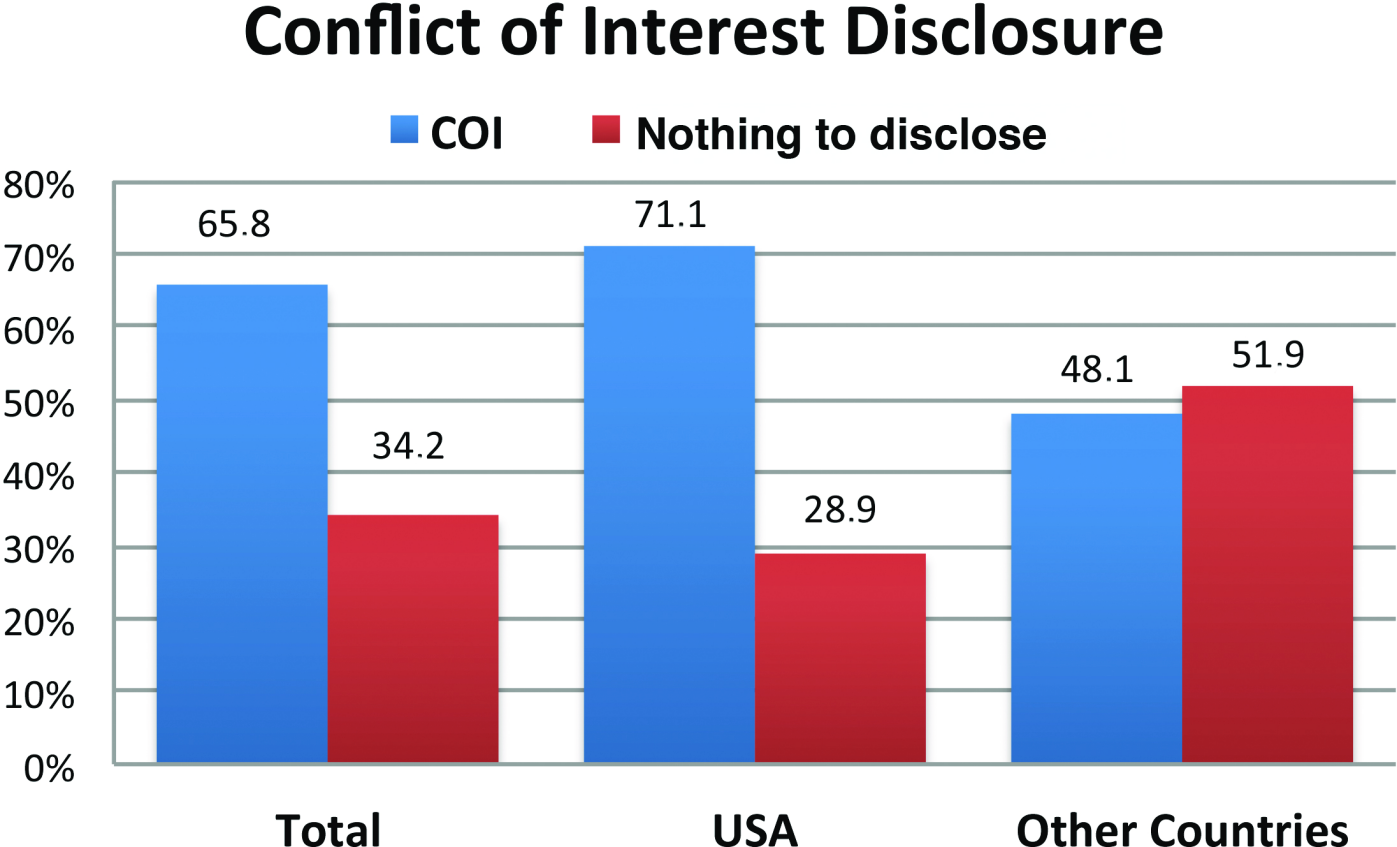
Potential COI disclosed by SRS and IMAST program review members (in percentages) corresponding to the period 2010–2014.

For the meetings analyzed, 77 of the 117 program reviewers (65.8%) reported a potential COI, whereas 40 (34.2%) reported nothing to disclose/no conflict. When reviewers were analyzed by origin, those from the United States disclosed COI at a significantly higher proportion than reviewers from other countries (Fisher’s exact test *p*<0.05) (Fig 1). Analysis of the whole sample reveals that the annual percentage of reviewers with COI ranged from 44.4% in 2012 to 80.0% in 2011. The annual percentage of members without a potential COI ranged from 20.0% (2011) to 55.6% (2012).

The program reviewers reported a total of 273 COI. The highest percentage corresponded to consultancy activities (36.0%), followed by research funds (26.1%). Table 1 shows the types of potential COI by reviewer origin. There were statistically significant differences between U.S. members and those from other countries in the disclosure of consultancy activities (*p* = 0.02), consultancy being more frequent in non-U.S. members. The mean ratio of disclosed COI to reviewers (of those who disclosed COI) was 3.54 (range per year: 1.62–3.93). The United States had a twofold greater COI to reviewer ratio than did other countries (Table 2).

**Table 1:** Potential conflict of interest. Data are given in number, and percentages in parentheses.

Conflict of Interest	Total Reviewers (n = 77)	USA (n = 64)	Other Countries (n = 13)
(a) Grants/ Research Support	71 (26.1)	66 (26.5)	5 (20.8)
(b) Consultant	98 (36.0)	84 (33.7)	14 (58.4)
(c) Stock/ Shareholder (self-managed)	24 (8.8)	24 (9.6)	-
(d) Speaker’s Bureau	29 (10.7)	24 (9.6)	5 (20.8)
(e) Advisory Board or Panel	29 (10.7)	29 (11.6)	-
(f) Salary, Contractual Services	2 (0.7)	2 (0.8)	-
(g) Other Financial Or Material Support (royalties, patents, etc.)	20 (7.4)	20 (8.0)	-
Total COI	273	249	24

**Table 2:** Number of potential COI and COI/authors ratio per year.

	Whole sample			USA			Other Countries		
	n	COI n	COI/reviewer	n	COI n	COI/reviewer	n	COI n	COI/reviewer
2010	22	74	3.36	19	69	3.63	3	5	1.66
2011	20	78	3.90	19	70	3.68	1	8	8.00
2012	8	13	1.62	4	9	2.25	4	4	1.00
2013	13	55	4.07	10	52	5.20	3	3	1.00
2014	14	53	3.93	12	49	4.08	2	4	2.00
Total	77	273	3.54	64	249	3.89	13	24	1.84

**Fig 2:**
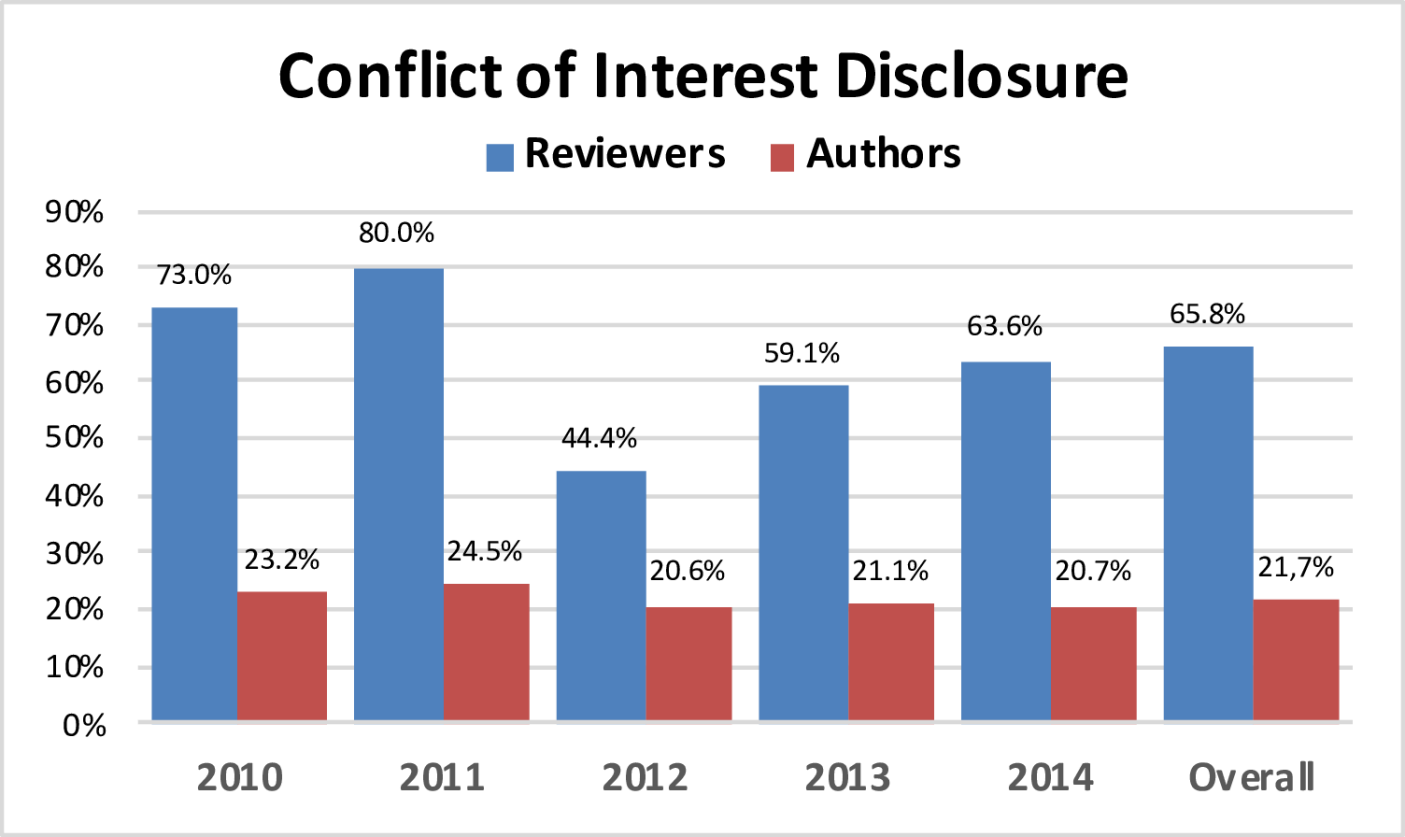
Distribution of COI (in percentages) by years differentiating reviewers and authors. (p<0.001 for each year and overall comparisons).

During the same 2010–2014 SRS-IMAST meetings, there was a total of 2728 authors excluding some of the reviewers that also participated in the oral presentations. Of these authors, a total of 592 disclosed COI (21.7%). The percentage of authors declaring COI showed small variations along the period of study, ranging from 20.6% in 2012 to 24.5% in 2011. Differences in COI declarations between program reviewers and authors we statistically significant along the years of study (p<0.001) (Fig 2). The number of COI was 2260 in total, with an overall mean ratio of disclosed COI to authors (of those who declared COI) of 3.82 (range per year: 3.30–4.12).

**Fig 3:**
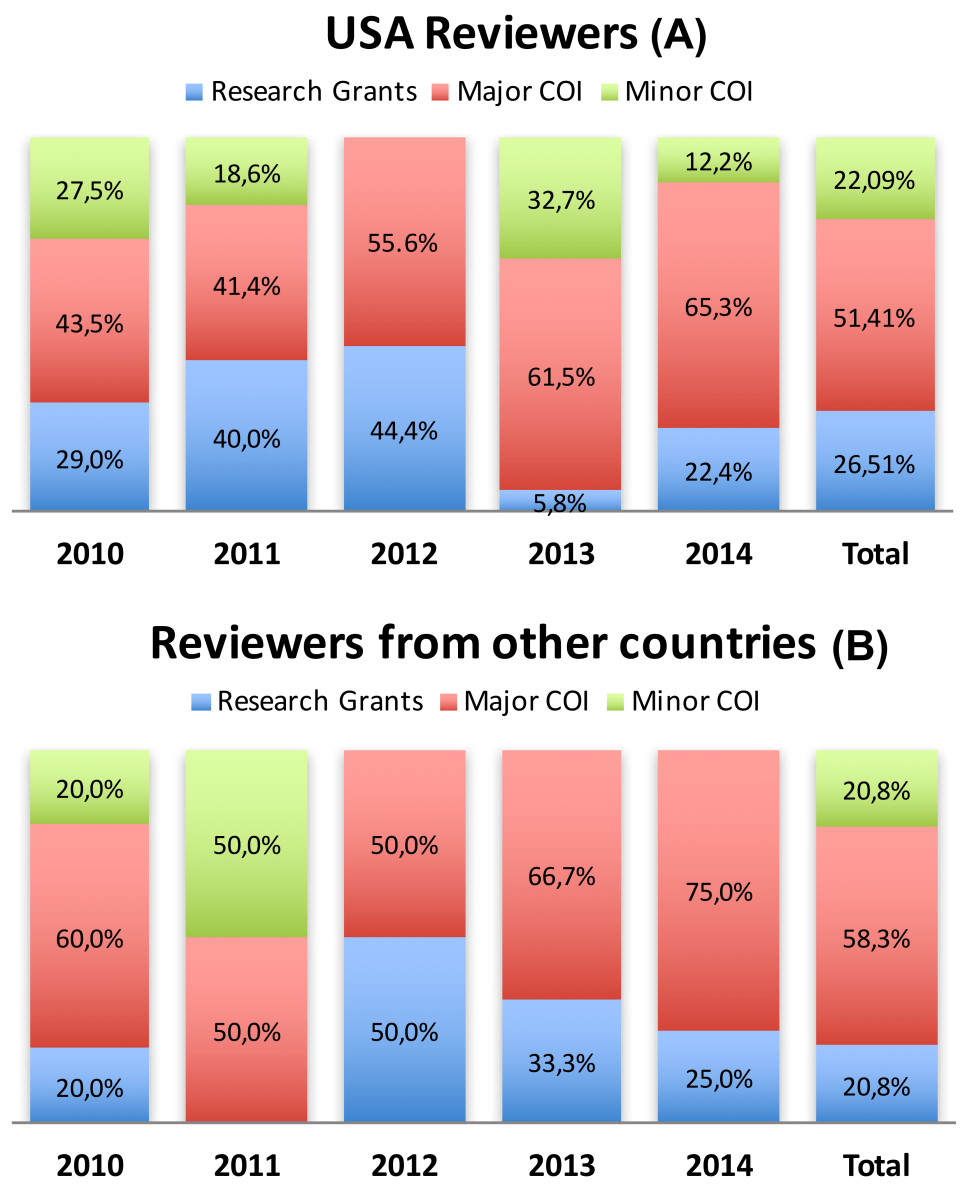
Type of COI disclosed by SRS and IMAST program review members along the period of study. (A) reviewers from the USA. (B) reviewers from other countries.

When consultant fees, stock options, and royalties were considered together as a major financial COI of reviewers, they accounted for 52.0% of COI. Minor COI (speakers bureau, advisory board or panel, and other contractual services) represented 22.0% of disclosures. Grants that support research were 26.0% of the reported COI. Fig 3A and Fig 3B show the distribution of major COI, minor COI, and research grants per year of study by reviewer origin. There were no differences between reviewers and authors in the type of COI declared. In the case of authors, the overall figures were: grants 29.8%, major COI 51.6%, and minor COI 18,6%.

**Fig 4:**
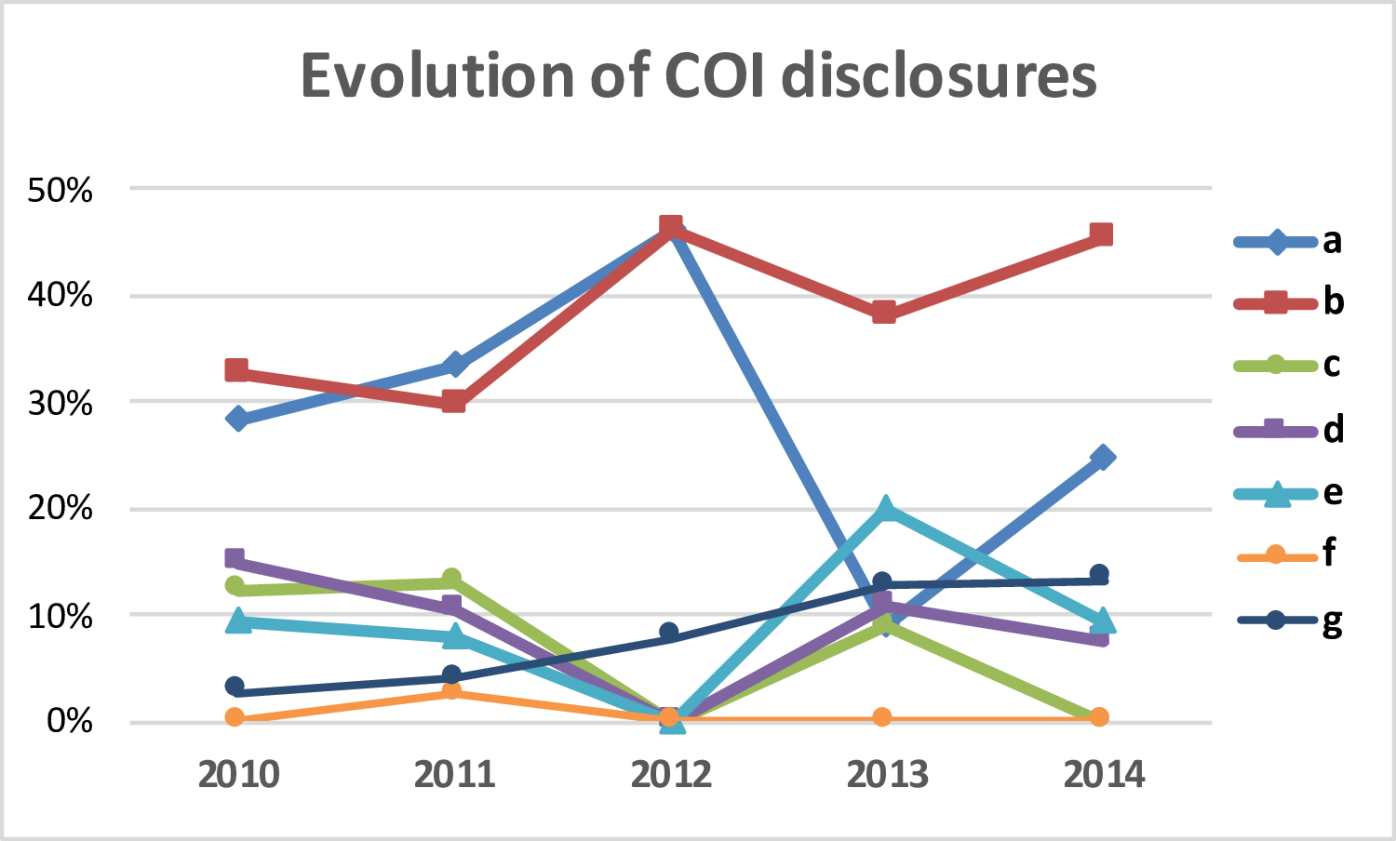
Evolution of the type of COI disclosures along the period of study. Type a) grant/research support; Type b) consultant; Type c) stock/shareholder (self-managed); Type d) speakers bureau; Type e) advisory board or panel; Type f) salary/contractual services; and Type g) other financial or material support (royalties, patents, etc.).

The evolution of COI declarations from 2010 to 2014 is shown in Fig 4. The most relevant findings were first the progressive increase of COI related to consultant activities, corresponding in 2014 to the 45.3% of all COI disclosures. A second observation was the reduction in the number of COI related to research grants along the 2010–2014 period. In 2013, research grants accounted only for the 9.1% of all disclosures, and for 24.5% in 2014. There was also a decrease in the number of disclosures related to speaker’s bureau activities (from 14.9% in 2010 to 7.5% in 2014). Grouping the different COIs, a continuous increase in the declaration of major COI was observed, starting at 47.3% in 2010 and ending at 58.5% in 2014. The percentage of minor COI slightly decreased from 24.3% in 2010 to 17.0% in 2014.

**Fig 5:**
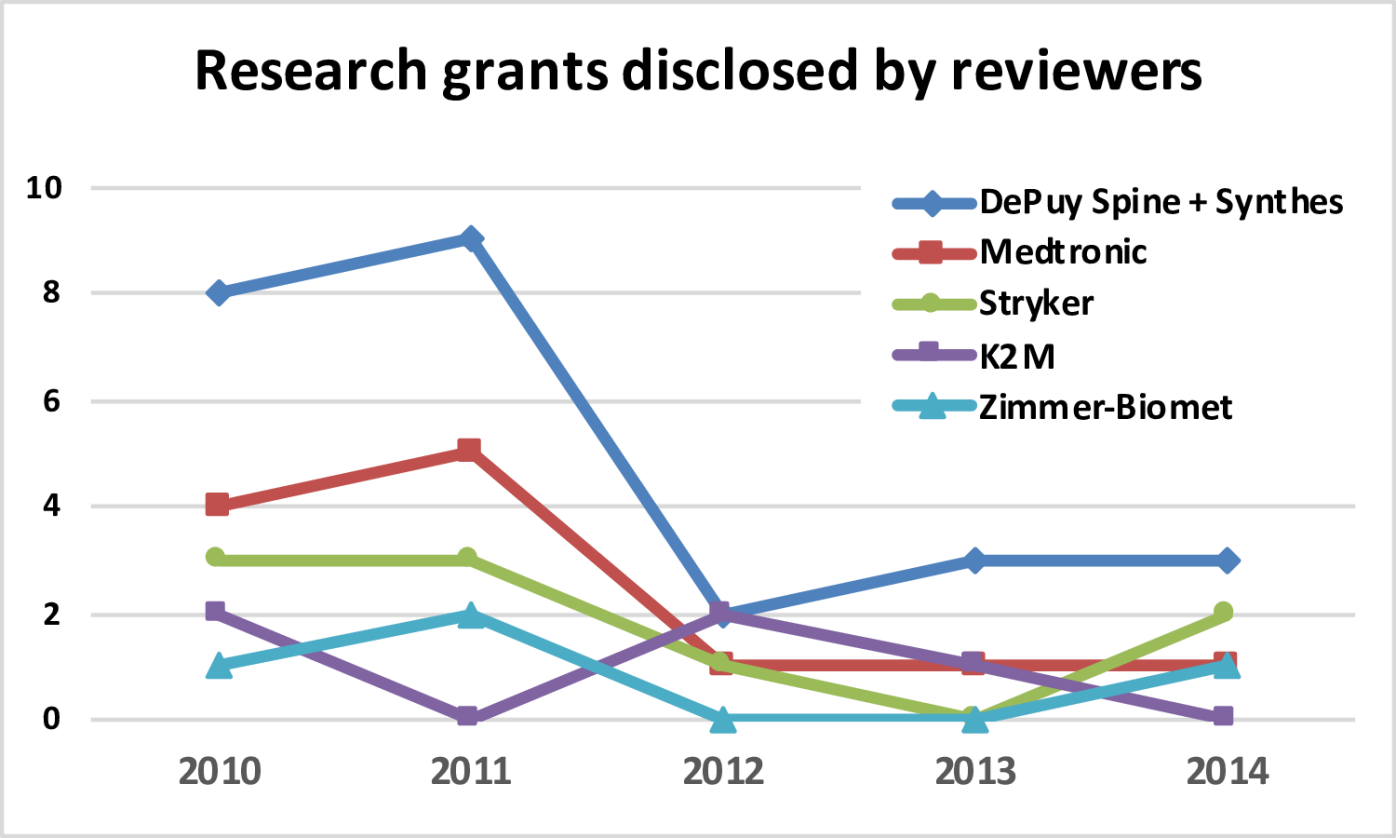
Number of research grants disclosed by the reviewers related to the leading spine market companies.

**Fig 6:**
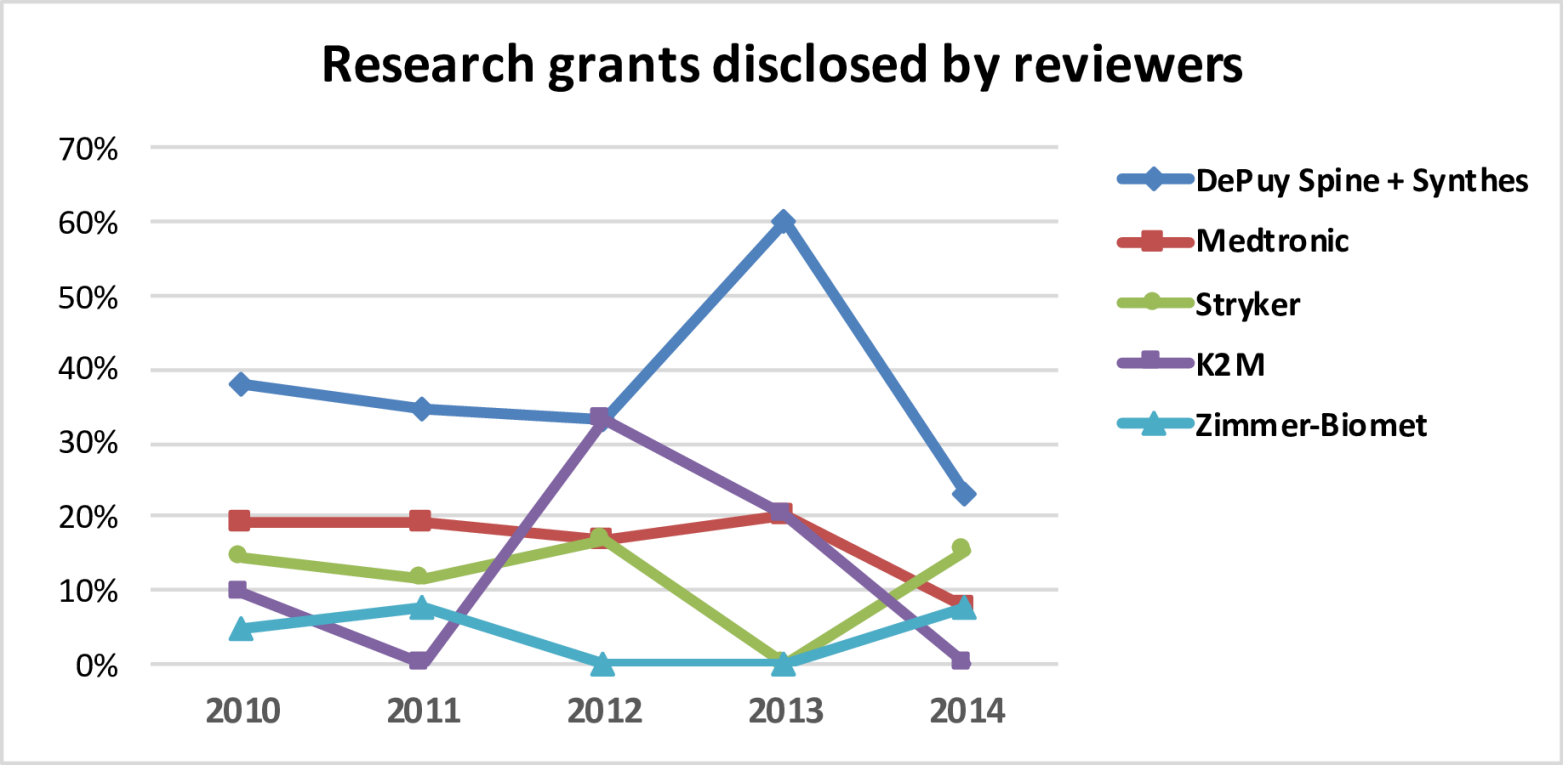
Distribution of research grants among the leading companies in relation to the total number of grants disclosed by the SRS and IMAST Program reviewers.

In the study period, 55 different companies were mentioned in COI disclosures. Five of the top companies in the global spinal implant and device markets were responsible for 65,2% of all disclosed COI. Eighteen companies were involved in research grants, with five of the leading companies overall accounting for more than two thirds of such COI (77.4%) (Table 3). The number of COIs related with research grants supported by the big five companies severely decrease in number after 2012 (Fig 5). The distribution of COI related to research grants among the big five companies during the 5-year period of study is summarized in Fig 6. DePuy Spine-Synthes lead this type of disclosed COI.

**Table 3:** Leading companies involved in the different COIs during the period 2010–2014.

	Total		Grants		Maior COI		Minor COI	
COI (n)	273		71		142		60	
Companies (n)	55		18		31		25	
COI/author/company	4.96		3.94		4.58		2.40	
Leading company	n COI	%	n COI	%	n COI	%	n COI	%
DePuy Spine + Synthes	73	26.7%	25	35.2%	35	24.6%	13	21.7%
Medtronic	45	16.5%	12	16.9%	24	16.9%	9	15.0%
Stryker	27	9.9%	9	12.7%	14	9.9%	4	6.7%
Zimmer Biomet	17	6.2%	4	5.6%	11	7.7%	2	3.3%
K2M	16	5.9%	5	7.0%	11	7.7%	-	-

**Fig 7:**
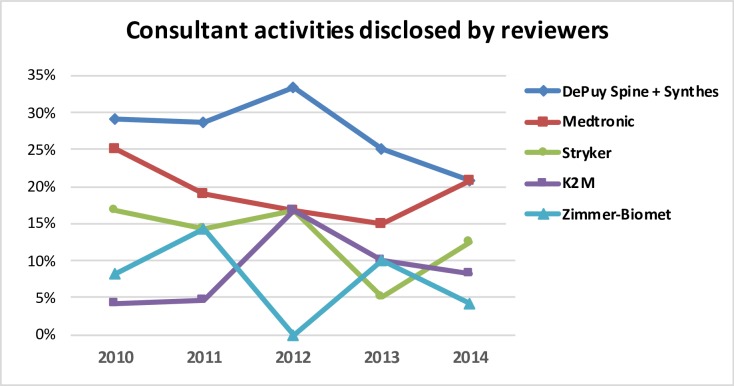
Distribution of consultant activities disclosed by the reviewers in relation to the total number of type b major COIs.

As for major COI, the same five leading companies were cited in 110 of the 142 COI disclosed (77.4%). Consultant activities related to the big five companies were more commonly disclosed among COI with major relevance (95 of 110; 86.4%). Fig 7 describes the consultant activities disclosed by the program reviewers as related to the five leading companies. The association of DePuy Spine and Synthes accounted for the highest number of major COIs related with consultant activities.

**Fig 8:**
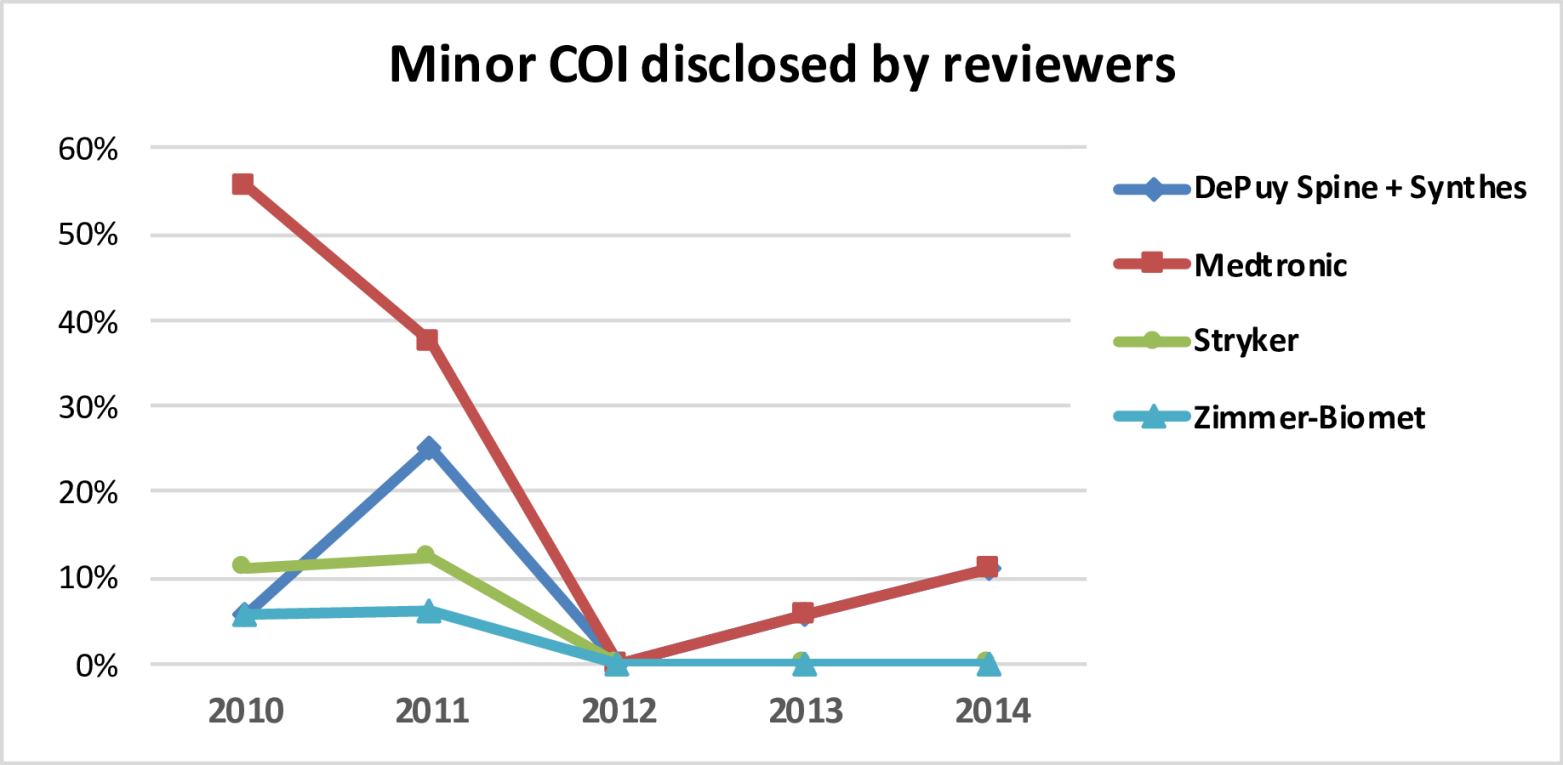
Distribution of minor COIs disclosed by the reviewers and associated to four of the five leading companies in relation to the total number of disclosed minor COI.

Minor COI were spread among 25 different companies, with four of the five leading companies overall involved in 51.6% of this type of disclosure. K2M was never referred among minor COI. An important change in the involvement of the big companies in. minor COI was observed along the period of study. Four big companies were responsible of 77.8% of the minor COIs in 2010, and 81.3% in 2011. However, the contribution of these four companies to minor COIs decreased notably in 2011 (11.8%) and in 2012 (22.2%). There were no minor COI related with these companies in 2012. Fig 8 shows the evolution of the minor COI related with the big companies. Most of the minor COIs corresponding to the period 2010–2012 were associated to Medtronic.

**Fig 9:**
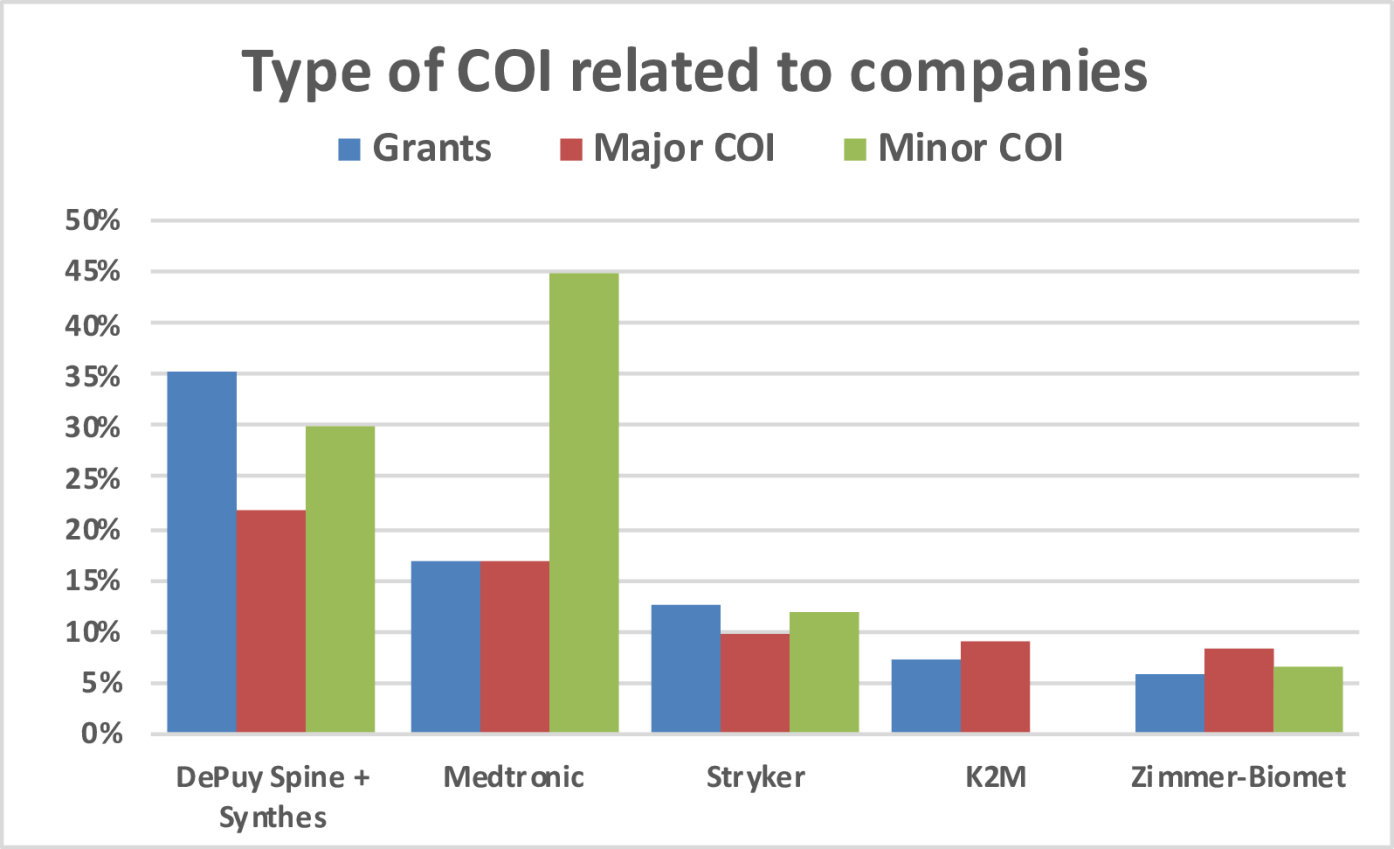
Overall distribution of COIs disclosed by the reviewers and related to the five leading companies.

Overall, the distribution of the type of COI declares by the program reviewers in relation to the five big companies of the spine implant market are shown in Fig 9. DePuy Spine-Synthes was the most frequent company related to research grants, and Medtronic in minor COI.

## Discussion

To our knowledge, this study is the first to examine the amount and type of COI disclosed by reviewers of the program committees of five consecutive SRS annual meetings. Importantly, this program committee also decides to accept or reject abstracts submitted to IMAST meetings. According to the disclosure statement index provided each year by SRS, almost two thirds (65.8%, 77 of 117) of reviewers declared potential COI, and more than half of the disclosures were considered as major COIs (consultant fees, stock options, royalties); these increased from 44.6% in 2010 to 66.0% in 2014. The mean disclosed COI per reviewer (of those who disclosed COI) was 3.54. These statistics reflect the strong interactions between industry and review program members.

When reviewers from USA were compared to those of the rest of the world, a significantly higher proportion of COI were declared by the USA reviewers (71.1% vs 48.1%). Interestingly, the percentage of reviewers declaring COI was significantly higher than that found in the authors participating in the different meetings. which never exceeded 24.5%. This disproportion between reviewers and authors could be understood by the fact that most of the reviewers use to be recognized surgeons with a longer professional career and, therefore, with more potential bounds to industry.

In 2008, Ju et al. [7] analyzed the author disclosure information published for the 2008 North American Spine Society (NASS), Cervical Spine Research Society (CSRS), and SRS. The percentage of COI disclosures among the authors that overlapped at least two of these three annual meetings varied from 24% to 36%. Contributing authors seem to have less COI than the reviewers analyzed in the current study. Regarding NASS meetings, a COI was reported for 10% of all presentations in 1985, increasing to 32% in 2002 [11]. In the 2006 SRS Annual Meeting, there were 27.9% of authors declaring COI [12]. Besides the rising of COI declaration by authors involved in spine research, the figures were always under those found for SRS program review members in the present study. The current data reflects the more frequent relationship to spine implants industry of the reviewers from USA as compared to those from other countries. In addition, USA reviewers that declared COI had a twofold greater COI/reviewer ratio than did reviewers from other countries (3.89 vs 1.84). The COI ratio for non-USA reviewers was certainly conditioned by a single reviewer of the total 13 that in 2011 declared 8 COI. The other 12 reviewers declared in total only 16 COI. A reason for the differences in the COI/reviewer ratio between USA and non-USA participants could be the different spine market coverage by the leading companies. The spine market world is currently led by companies from USA. These top companies could have less implantation in some of European and Asiatic countries where the non-USA reviewers had their origin.

A potential explanation for these high rates of industry relationships of SRS program reviewers is the relatively small nature of the scoliosis community and the field’s heavy reliance on implant technology. Advances in the surgical treatment of spinal deformities are usually related only to surgeon expertise. As such, the industry must develop new technologies. Furthermore, as reputed spine surgeons, a significant number of review program members unsurprisingly show interactions with industry, resulting in frequently unavoidable COI.

This is the first study analyzing separately the COI of meeting program reviewers. Therefore, comparisons with other studies are not possible. In the current study, the percentage of spinal surgery program reviewers with potential COI (65.8%) is substantially higher than that found for reviewers of spine journals (29%) [9]. Obviously, journals are different than meetings, but the function of the reviewers is quite similar: the acceptance of manuscripts to the journal or abstracts for presentation at the meetings. The authors of the study concerning COI editorial board members of spinal journals believe they likely underestimated the reported figures, as they were only able to evaluate half of all editorial board members [9]. The present study had complete access to published COI data, and therefore the results describe a quite real vision of the industry relationships of the SRS-IMAST program review members.

One interesting finding is the progressive increment of major COI along the period of study (58.5% in 2014), especially those related to consultant activities. In the last year analyzed, consultant activities corresponded with almost half of the declared COI. Analyzing the frequency and type of COI declared by authors in the 2006 SRS annual meeting, grants accounted for 51.1% of the disclosures and consultant relationships only for the 20.8% [12]. The increment in major COI of SRS program reviewers overlaps with a reduction in the number of minor COI and those related to research grants. The reasons are unknown from outside the companies. These findings indicate that the policy of companies concerning relationships with spine surgeons are progressively changing. More specifically, this data reflects that the interactions of reviewers with industry are more intense over time.

Regarding the source of COI, two thirds of the declared relationships were related with only five big companies covering the spine implant market. Considering only major COI, 86.4% of the disclosures were due to consultant activities for the five top companies. Three of these companies were not involved in minor COI after 2012. according to the current data, five big companies are supporting most of the research and consultant activities of the SRS program reviewers. These findings are similar in arthroplasty research. Authors presenting arthroplasty-focused research at the 2012 annual meeting of the American Academy of Orthopaedic Surgeons (AAOS) and declaring COI reported also paid consultancy (51.5%), and research support (43.0%) from industry [13]. According to a previous study, research in surgical correction of spinal deformities has very little prospect for NIH funding, despite the importance of the topic [12]. The lack of government support has been taken over by the industry. Nevertheless, in this scenario, to maintain the independence from industry should be a very complicate matter for program reviewers.

These interactions can have both beneficial and negative effects. On one hand, ties between the spinal device industry and reviewers could be essential for sharing state-of-the-art scientific knowledge and advancing spinal surgery. On the other hand, these strong financial bonds might harm the impartiality of the review process to a certain extent, resulting in unintentional selection bias [14,15]. These are not mutually exclusive effects.

The process of deciding which abstracts are presented at scientific meetings is certainly a source for concern among medical societies. As have other scientific societies, the SRS has made a great and commendable effort to ensure that abstract selection not be biased by potential COI among program review members. The SRS CME Committee is one of the mechanisms that guarantee the ethical integrity of this process. However, the process could likely be improved by publishing the names of program committee members together with their potential COI before the submission process. The scientific society’s board of directors could contemplate the possibility that some authors may reject reviewers at the time of abstract submission because of COI disclosed by those reviewers. This might reinforce the value of COI disclosure requirements.

Medical associations should be aware of the potential bias that could be caused even unintentionally by program reviewers and should strive to improve transparency during the selection process. Current recommendations proposed for medical journals to avoid altered conclusions or outright rejection of submitted manuscripts could be adapted and incorporated by program review committees of scientific meetings [9,14–18]. Some or all of the following suggestions could be implemented by scientific societies in order to improve the transparency of the review process. First, scientific societies should consider developing specific COI policies for program review committee members. Second, program reviewers and their potential COI should be announced before abstract submission. These lists could be published online. Third, scientific societies should allow authors to decline a review by certain members based on their potential COI, just as scientific journals do. Last, the board of directors could select some reviewers who have no relationships with medical device or implant companies. These independent reviewers could assess the scientific and methodological data of submissions. All of these suggestions aim to minimize bias in the abstract review process.

The scientific method attempts to make science as free from bias as possible [19,20], but bias is in some ways unavoidable. Physicians with conflicts of interest should not necessarily be disqualified as good reviewers. Similarly, reviewers who receive research grants from industry should not necessarily become ineligible to judge the merits of papers on bracing, basic science, or postoperative pain management.

This study is not free from limitations; therefore, the findings should be interpreted carefully. The most obvious limitation is that the financial COI extracted from the index published by SRS were self-reported, which implies irregularities, inconsistencies, and inadequate disclosures. This limitation was demonstrated in a relatively recent study that quantified the variability in self-reported disclosures of individual authors who presented at three of the leading spinal conferences (NASS, CSRS, and SRS) held in 2008 [7]. Among authors presenting at the NASS and CSRS meetings, 51% exhibited discrepancies in their disclosure information. In contrast, only 9% of authors whose COI disclosures were indexed at both the NASS and SRS conferences demonstrated irregularities. Similarly, 18% of authors presenting at both the CSRS and SRS conferences were inconsistent in their reporting. Although these data only examine presenters’ COI, program reviewers are similarly susceptible to this variability in COI reporting because they have to follow the same guidelines as authors.

## Conclusions

These results confirm that members of the SRS and IMAST scientific committees have a high percentage of COI. Two thirds of the reviewers (65.8%) disclosed COI, which is high compared to authors (27.1%). The average of disclosed COI was 3.54 per reviewer who declare COI. Consultancies and research grants account for two thirds of COI. Most of the grants and major COI are related to five of the leading companies in the spinal implant market. As expected, this study shows that SRS review program members have significant relationships with the spine implants industry. The diagnosis and treatment of spine diseases and deformities are highly dependent on new technologies; therefore, relationships with industry are completely understandable, not necessarily unethical, and unavoidable in many instances. Scientific societies should minimize the potential bias that COI disclosed by program reviewers could introduce during the abstract selection process. To improve transparency in that process, scientific societies should select as many reviewers as possible without relationships with medical device or implant companies. These independent reviewers could assess the scientific and methodological data of submissions. All of these suggestions aim to improve transparency in the abstract review process and scientific independency from industry.
